# Milk Hygiene in Rural Southwestern Uganda: Prevalence of Mastitis and Antimicrobial Resistance Profiles of Bacterial Contaminants of Milk and Milk Products

**DOI:** 10.1155/2017/8710758

**Published:** 2017-01-26

**Authors:** Paul Ssajjakambwe, Gloria Bahizi, Christopher Setumba, Stevens M. B. Kisaka, Patrick Vudriko, Collins Atuheire, John David Kabasa, John B. Kaneene

**Affiliations:** ^1^College of Veterinary Medicine, Animal Resources and Biosecurity, Makerere University Kampala, P.O. Box 7062, Kampala, Uganda; ^2^Medical Research Council, Uganda Virus Research Institute, P.O. Box 49, Entebbe, Uganda; ^3^MTK Uganda Ltd., P.O. Box 924, Nasser Road, Kampala, Uganda; ^4^Department of Health Sciences & Special Education, Africa Renewal University (AfRU), P.O. Box 35138, Kampala, Uganda; ^5^Center for Comparative Epidemiology, Michigan State University, 736 Wilson Rd., Room A-109, East Lansing, MI 48824, USA

## Abstract

Mastitis and antimicrobial resistance are a big challenge to the dairy industry in sub-Saharan Africa. A study was conducted in Kashongi and Keshunga subcounties of Kiruhura District (in Uganda) where the government and private sector have deliberate programs to improve production efficiency, quality, and safety of milk and its products. The study aimed to determine the prevalence of mastitis, its common causative agents, antimicrobial sensitivity of mastitis causing organisms, and contaminants of processed milk products: yoghurt and ghee. Seventy-one milk, fourteen yoghurt, and three ghee samples were collected from nine farms. Of the 71 cows tested, 54 (76.1%) had mastitis. The mastitis cases from Keshunga were 32 (59.3%) and Kashongi contributed 22 (40.7%) of the cases. The common mastitis causative agents were* Staphylococcus *spp. (30.8%),* Streptococcus *spp. (12.3%),* Corynebacterium* spp.(15.4%), and* E. coli *(7.7%). Some of the isolates were resistant to tetracycline and penicillin. Prevalent contaminants of yoghurt were* Staphylococcus *spp. (8.3%),* Streptococcus *spp. (8.3%),* Corynebacterium *spp. (8.3%), and* E. coli *(8.3%), whereas all ghee contained* Streptococcus *spp. (100%). Prevalence of mastitis, antimicrobial resistance, and contamination of milk products are high in the study area. Targeted programs to prevent and control mastitis as well as antibiotic resistance are recommended.

## 1. Introduction

Over the years, the global meat and milk production has increased in response to an expanding demand [1]. Similarly, livestock production is also on the increase in Uganda. Dairy farming has sustainably exhibited high growth since the late 1980s, especially in the western part of the country. About one-third (1.7 million) of the households in Uganda keep cattle as a source of income, nutrition, and employment. The cattle population in Uganda is 11.4 million, of which 93.6% were indigenous breeds, 5.6% were dairy cattle of exotic or cross-breed, and only 0.8% were of exotic or cross-breed beef cattle. The annual milk production is 1.5 metric tonnes [2]. This is still low since it can only satisfy 20% of the nutritional needs of the entire population. The consumption of milk per capita in Uganda has increased over the last decade and is about 50 litres per year [3, 4].

In terms of consumption, approximately 30% of the milk is consumed by the producing households, 70% is sold in markets, and between 10 and 20% of the marketed milk is processed into different dairy products. The rest is sold informally as raw milk. However, as much as there is gradual improvement in milk production, the dairy sector is faced by many challenges including disease conditions, such as mastitis [5–7]. Mastitis is still a very important animal welfare and economic problem in dairy cattle production systems. It presents with significant economic implications, such as drop in production, compromised milk quality, and extra costs on veterinary services [8]. Though it can be treated, the needed professional advice is not readily available or adhered to, especially in sub-Saharan Africa where veterinary service provision is fragmented [9]. As a result of these shortfalls, this condition is still prevalent and its related risks of antibiotic resistance have been reported [10]. This is because there is a lot of injudicious use of drugs by farmers who attempt to manage the condition with limited professional guidance. This study set out to determine the prevalence of mastitis and its associated microbial agents and to assess their possible drug resistance against the available therapeutic agents on the market. This may form a basis to advance appropriate extension advice to farmers, local veterinarians, and the other relevant stakeholders, as well as open areas of further research [11].

## 2. Materials and Methods

### 2.1. Study Area and Design

This cross-sectional study was carried out in the subcounties of Kashongi and Keshunga in Kiruhura District, located in Southwestern Uganda. Thus, the AgShare project catchment area included farms, milk collection centers, and local milk product processing plants/homesteads. Purposive sampling techniques were used to include only farms that had milking cows. Consecutive sampling was used to select the milk collection centers where farmers sold the milk. Selection of points of sale of milk products was by convenience.

### 2.2. Ethical Consideration

Participating farmers were given full and adequate oral and written information about the nature, purpose, possible risks, and benefits of the study in both English and Runyakitara languages. They were given adequate opportunity to ask questions and allowed time to consider the information provided. The participants signed and dated informed consent that was obtained before conducting this study. The study data were stored in a computer database while maintaining confidentiality. Participating farms in this database were identified by the unique enrolment number only. Ethical clearance to carry out this study was sought from the Research and Ethics Committee of the School of Veterinary Medicine at Makerere University and Kiruhura District Administration.

### 2.3. Sample Collection and Handling

Using the California Mastitis Test (CMT) technique by Mellenberger and Roth [12], with minor modifications as indicated, a four-compartment paddle was introduced below the udder of a milking cow, after having let out the fore milk. Approximately two millilitres of milk was drawn from each quarter onto the paddle. An equal volume of CMT reagent was added, followed by gentle swelling of the paddle. The results were read after 15 seconds. Of the positive mastitis cases, a corresponding milk sample was collected for laboratory analyses as described in the next paragraph. We obtained 5 mL of milk from the udder from at least 2 different quarters. This was milked directly into sterile plastic tubes, and the samples were then stored in cool-freezer boxes containing ice blocks and transported to the laboratory for analysis within 72 hours after collection.

### 2.4. Bacterial Culture and Isolation

Milk samples were serially diluted in 1% peptone water before plating onto Mannitol Salt Agar (MSA, Oxoid®) to select for* Staphylococcus aureus*, Violet Red Bile Agar (VRBA, Oxoid) for coliforms, Plate Count Agar (PCA, Oxoid) for aerobic spore formers, and Oxytetracycline Glucose Yeast Extract Agar (OGYE®) for any yeast or moulds. Inoculated plates using the streaking method were incubated at 37°C for a maximum period of 48 hours. Bacterial colonies were identified using their common phenotypic characteristics. Additionally, conventional biochemical methods were applied to the isolates for further identification.

### 2.5. Gram Staining

Each bacterial isolate was picked using a sterile loop and smeared onto a clean microscope slide and then air dried. This smear was fixed using the flaming method and thereafter flooded with crystal violet solution and left to stand for 1 minute. The smear was then briefly washed with distilled water and then flooded with Gram's iodine solution and left to stand for 1 minute and thereafter washed with distilled water and decolorized using 95% alcohol. This was subsequently washed with distilled water, followed by flooding with safranin counter stain for 50–60 seconds. The smear was washed, blot dried, and examined under the microscope. Gram positive bacteria stained blue to purple and Gram negatives stained pink to red [13].

### 2.6. Antibiotic Sensitivity Testing

Using the disc diffusion method, with modifications as indicated [14], inocula of isolated strains were prepared in 1% peptone water and adjusted to turbidity equal to 0.5 McFarland standard and then applied onto Mueller Hinton sensitivity agar using a sterile wire loop by streaking on the agar surface. A sterile swab was used to spread the culture solution onto the solid media. The inoculated plate was left to dry in a biosafety cabinet for 10 minutes. Thereafter, antibiotic sensitivity discs were applied onto the inoculated agar using sterile forceps and placed in a 37°C incubator. Zones of inhibition were measured after 18–24 hours of incubation. The antibiotics used were penicillin (10 *μ*g), tetracycline (30 *μ*g), gentamycin (10 *μ*g), streptomycin (10 *μ*g), neomycin (10 *μ*g), and cotrimoxazole (10 *μ*g).

### 2.7. Data Handling and Analysis

The collected data were recorded in prepared data sheets. Data were entered in MS Excel spreadsheets and exported to SPSS (version 20, IBM®) for statistical analysis.

## 3. Results

The study involved five farms in Keshunga and four farms in Kashongi subcounties, respectively. In total, 71 milking cows were sampled by taking milk from at least 2 quarters of the udder. Of the 71, 37 were from Keshunga and 34 were from Kashongi. All were cross-breeds of Friesians. Milk products that were assessed included yogurt (14 samples) and ghee (3 samples).

### 3.1. Prevalence of Mastitis in Kiruhura District

**Figure 1:**
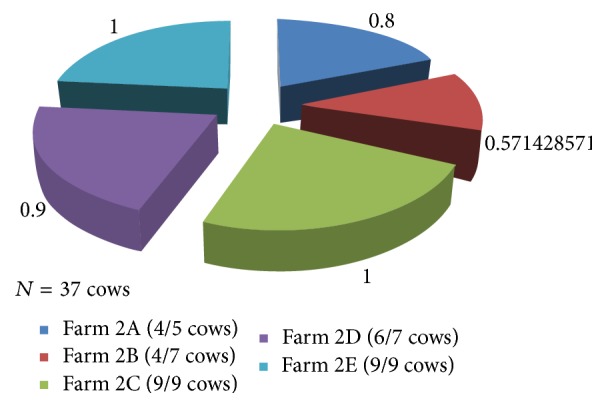
Proportion of cows that tested positive for mastitis on each farm in Keshunga subcounty.

**Figure 2:**
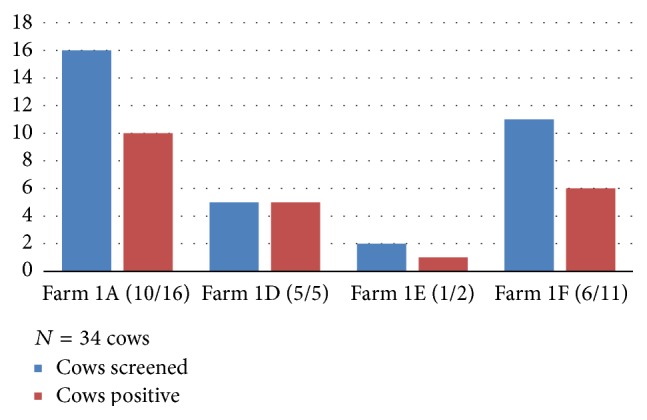
Proportion of cows that tested positive for mastitis on each farm in Kashongi subcounty.

Of the 71 cows tested, 54 (76.1%) were positive to mastitis. The mastitis cases from Keshunga were 32 (59.3%), whereas Kashongi contributed 22 (40.7%) of the cases. On some farms in both subcounties, all milking cows sampled had mastitis. Other details of mastitis on each farm are shown in Figures 1 and 2. Please note that all positive cases were subclinical mastitis.

**Figure 3:**
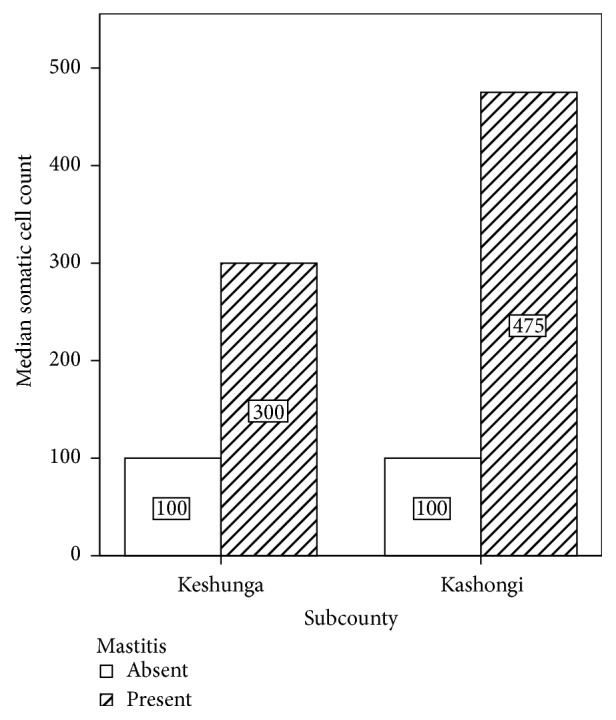
Burden of mastitis in terms of somatic cell counts (SCC) per subcounty.

In terms of somatic cell counts (SCC), the burden of mastitis was greater in Kashongi (median SCC = 475) compared to Keshunga (median SCC = 300). This is regardless of the fact that the former had fewer mastitis cases (22) than the latter (32). However, this difference was not statistically significant (*p* = 0.056). The details of this comparison are shown in Figure 3.

### 3.2. Bacterial Isolates Associated with Mastitis and Contamination of Milk Products

In the milk samples,* Staphylococcus* were the most common bacteria with a prevalence of 30.8%, followed by* Proteus* at 13.8%. In the yoghurt samples,* Bacillus* was the most prevalent organism. Ghee samples only had* Streptococcus*. Other details are shown in Table 1.

### 3.3. Antimicrobial Susceptibility Profiles of Bacteria Isolated from Milk and Milk Products

**Figure 4:**
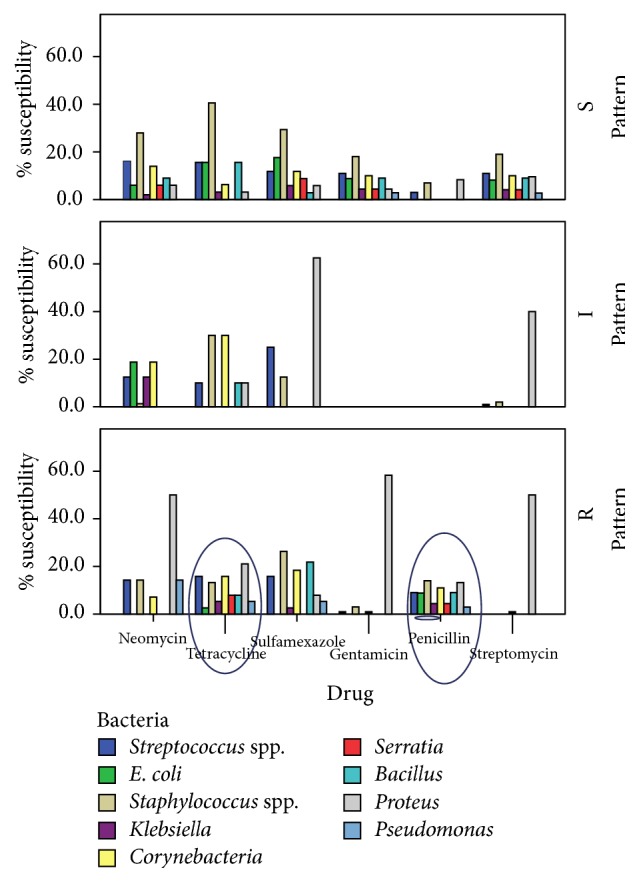


As shown in Figure 4, bacteria such as* Streptococcus *spp.,* Staphylococcus *spp., and* E. coli *showed resistance to tetracycline. Furthermore, a similar observation was noted against penicillin for the same isolates of bacteria. Other details are shown in Figure 4.

## 4. Discussion

The study district had a significantly high burden of mastitis in the two subcounties among the sampled farms. Some of the common practices in this area that did predispose to mastitis are deficient dry cow therapy, unhygienic milking practices (such as sharing the same milking towel among different cows), milk men not washing their hands with clean water before milking and in between milking procedures of different animals, and the absence of a milking parlor, among others, as described by Kaneene et al. [11] and Abrahmsén et al. [7]. Persistence of these factors in the area may be attributed to high costs associated with practicing some of the recommended activities that contribute to reduced mastitis prevalence. In addition, the high prevalence of mastitis has been reported to be due to poor udder hygiene, high yielders, and zero grazing [10].

The most prevalent organisms associated with mastitis were* Staphylococcus *spp.*, Streptococcus *spp.*, Corynebacterium*, and* E. coli,* among others*. Staphylococcus *spp. are common skin flora [15], and once hygiene practices during milking, such as using a clean hand towel per animal to clean the teats and postmilking teat dipping, are not implemented, the bacteria gain entry into the udder.* Staphylococcus *spp. have been reported in earlier studies as a causative agent of subclinical mastitis [10, 16]. In addition, when the immune system of the host is compromised, the originally normal skin flora establishes an infection leading to mastitis cases. The same applies to* Streptococcus *spp. once they access the teat canal and establish themselves in the udder, and they could also cause mastitis as reported in earlier studies.* Corynebacterium *spp. are another significant causative agent for mastitis and have also been documented by Watts et al. [17].

Mastitis cases due to* E. coli* are an indicator of poor milking hygiene [18] as this bacterium is a normal flora in the digestive tract and thus an indicator of fecal contamination as reported by Mellenberger and Kirk [19]. Furthermore, a practice in the study community that could contribute to this was the smearing of the teats of milking animals with cow dung to prevent weaner calves or heifers from suckling the dam or milking cows while in the fields. This vice comes with cost implications.

Although value addition is a much recommended practice for farmers and other stakeholders to harvest more revenue from processed milk products, safety of the produce is paramount. Presence of potential pathogens in yoghurt and ghee as seen in Table 1 is undesirable, posing health related risks to the consumers. This could also be an indication that hygienic practices were not adhered to during processing and storage. In a study by Salo et al. [20], it was noted that storage of milk at low temperatures minimizes the growth of microbes that could be potential pathogens to the consumers and also spoilers of the milk and its products. With this being said, storage facilities with low temperatures were not readily available in the study area and this could explain the presence of some of these microbes in milk and its products from this study. Furthermore, in a survey by Wong [21], it was shown that* B. cereus* was found in 52% of ice creams, 35% of soft ice creams, 29% of milk powders, 17% of fermented milk, and 2% of pasteurized milk and fruit-flavoured milk. This is a justification to have checks for potential pathogenic bacteria in milk products for quality and safety of these ready-to-consume foods as an important biosafety measure. In addition, it was observed that all the ghee samples for this study had* Streptococcus *spp. This could be explained by the fact that* Streptococcus *spp., especially* Streptococcus salivarius*, are among the lactic acid bacteria, important during the ghee fermentation process for Ugandan ghee [22].

In the study area, some of the common antibiotics used by farmers to control some infections are tetracycline and penicillin (Hytet® and PenStrep®, resp.), bought over the counter and used without the recommended veterinary supervision. Having in mind that these antibiotics are broad-spectrum, the farmers use them uncontrollably to treat a range of infections. Findings in this study (Figure 3) show that the above antibiotics did not inhibit growth of some isolates to which they should have been responsive. Simple practices, such as the use of one clean warm water soaked hand towel per animal, milking mastitis cows last, teat dipping with appropriate disinfectants, and the use of dry cow therapy, can significantly control mastitis in a herd. Antibiotic therapy is best recommended in clinical mastitis cases as compared to subclinical mastitis with professional guidance from a trained veterinarian.

The challenge is that veterinary services are not readily available, so farmers do carry out self-medication. As the required dosages are not always met, chances of development of chronic cases and/or antibiotic resistance are not uncommon [10, 23]. In a study by Soriano et al. [24],* Corynebacterium *spp. were shown to be susceptible to tetracycline; however, in this study, resistance to this drug was noted. The development of resistance could be as a result of the pathogen having adopted mechanisms at molecular level to evade the drug target pathways in the known pharmacology to effect its inhibitory or bactericidal roles [25]. The aforementioned practices may explain the existing antibiotic resistance. The study was cross-sectional in design and this did not allow assessment of the effect of seasonal variations on mastitis. We also did not investigate the risk factors associated with the observed levels of mastitis and antibiotic resistance. Due to the limited budget, our study did not assess the physiological, immunological, and nutritional status of the animals tested. However, the general practice was to feed the animals on pastures in the fields in both districts. One further limitation of our study (due to budget limitation) is the inadequate and imbalanced sample size. The results thus should be interpreted in light of these limitations.

We conclude that mastitis is still a major challenge in the study area with the most common associated agents being* Staphylococcus aureus*,* Streptococcus *spp.,* Corynebacterium *spp., and* E. coli.* Tetracycline and penicillin were among the drugs with the highest resistance observed to the bacterial isolates. We recommend that ongoing extension programs on dairy production be redesigned and expanded to include prevention and control of mastitis, as well as address antibiotic resistance.

## Figures and Tables

**Table 1:** Sources of the different bacterial isolates.

		Type of sample		
Bacteria	Milk, *n* (%)	Yoghurt, *n* (%)	Ghee, *n* (%)	Total, *N* (%)
*Staphylococcus*	20 (30.8%)	1 (8.3%)	0 (0%)	21 (26.3%)
*Streptococcus*	8 (12.3%)	1 (8.3%)	3 (100%)	12 (15%)
*Corynebacterium*	10 (15.4%)	1 (8.3%)	0 (0%)	11 (13.8%)
*Proteus*	9 (13.8%)	1 (8.3%)	0 (0%)	10 (12.5%)
*Bacillus*	5 (7.7%)	4 (33.3%)	0 (0%)	9 (11.3%)
*E. coli*	5 (7.7%)	1 (8.3%)	0 (0%)	6 (7.5%)
*Klebsiella*	3 (4.6%)	0 (0%)	0 (0%)	3 (3.8%)
*Serratia*	3 (4.6%)	0 (0%)	0 (0%)	3 (3.8%)
*Pseudomonas*	1 (1.5%)	1 (8.3%)	0 (0%)	2 (2.5%)
*Lactobacillus*	1 (1.5%)	0 (0%)	0 (0%)	1 (1.3%)
Mucoid *E. coli*	0 (0%)	1 (8.3%)	0 (0%)	1 (1.3%)
Nonmucoid *E. coli*	0 (0%)	1 (8.3%)	0 (0%)	1 (1.3%)
